# Functions of regulators of G protein signaling 16 in immunity, inflammation, and other diseases

**DOI:** 10.3389/fmolb.2022.962321

**Published:** 2022-09-02

**Authors:** Miaomiao Tian, Yan Ma, Tao Li, Nijin Wu, Jiaqi Li, Huimin Jia, Meizhu Yan, Wenwen Wang, Hongjun Bian, Xu Tan, Jianni Qi

**Affiliations:** ^1^ Shandong Provincial Hospital Affiliated to Shandong First Medical University, Jinan, China; ^2^ Zibo Central Hospital, Zibo, China; ^3^ Shandong Provincial Engineering and Technological Research Center for Liver Diseases Prevention and Control, Jinan, China

**Keywords:** RGS16, GPCR, immunity, inflammation, tumor, metabolic disorders

## Abstract

Regulators of G protein signaling (RGS) act as guanosine triphosphatase activating proteins to accelerate guanosine triphosphate hydrolysis of the G protein α subunit, leading to the termination of the G protein-coupled receptor (GPCR) downstream signaling pathway. RGS16, which is expressed in a number of cells and tissues, belongs to one of the small B/R4 subfamilies of RGS proteins and consists of a conserved RGS structural domain with short, disordered amino- and carboxy-terminal extensions and an α-helix that classically binds and de-activates heterotrimeric G proteins. However, with the deepening of research, it has been revealed that RGS16 protein not only regulates the classical GPCR pathway, but also affects immune, inflammatory, tumor and metabolic processes through other signaling pathways including the mitogen-activated protein kinase, phosphoinositide 3-kinase/protein kinase B, Ras homolog family member A and stromal cell-derived factor 1/C-X-C motif chemokine receptor 4 pathways. Additionally, the RGS16 protein may be involved in the Hepatitis B Virus -induced inflammatory response. Therefore, given the continuous expansion of knowledge regarding its role and mechanism, the structure, characteristics, regulatory mechanisms and known functions of the small RGS proteinRGS16 are reviewed in this paper to prepare for diagnosis, treatment, and prognostic evaluation of different diseases such as inflammation, tumor, and metabolic disorders and to better study its function in other diseases.

## Introduction

G protein-coupled receptors (GPCRs) are the largest superfamily of membrane proteins that control most cellular signaling and regulate key biological functions, including immune, inflammatory, oncological, and metabolic processes, by coupling to G proteins to transmit extracellular signals into the cell (Syrovatkina et al., 2016). In the resting state, heterotrimeric G proteins, which consist of α, β and γ subunits, bind to guanosine diphosphate (GDP). (Neves et al., 2002). In the state of external stimulation, GPCRs act as guanine nucleotide exchange factors (GEFs), facilitating the exchange of guanosine triphosphate (GTP) with GDP on Gα, and the activated GTP-Gα dissociates from the Gβγ dimer, undergoing conformational changes and regulating downstream effector proteins (Gilman, 1987). The α subunit is enzymatically active, and can catalyze the hydrolysis of GTP to GDP, after which Gα reassociates with Gβγ and returns to the resting state. Depending on the structural and functional differences, Gα subunits, including Gs, Gi/o, Gq/11, G12/13, etc., can mediate different signaling pathways (Soundararajan et al., 2008).

The regulators of G protein signaling (RGS) proteins, , which were discovered at the end of the 20th century, are a family of molecularly diverse and multifunctional proteins and are capable of binding to G protein-activated α-subunits, activating guanosine triphosphatases (GTPases) and accelerating the hydrolysis of GTP (>1,000-fold), thereby terminating the G protein signaling pathway (Koelle, 1997). Dysregulation of RGS expression is involved in a variety of diseases, including cancer, and cardiovascular and neurodegenerative diseases (Alqinyah and Hooks, 2018). According to the homology of the amino acid sequence and the different external signal domains, the typical RGS proteins can be divided into four groups: A/RZ, B/R4, C/R7 and D/R12 (Du and Huang, 2005).

Members of the B/R4 subfamily include RGS1-5, 8, 13, 16, 18 and 21, which are the smallest RGS proteins except for RGS3 (Bansal et al., 2007). RGS16, also known as A28-RGS14 or RGS-R, is expressed in a variety of tissues, such as the retina, pituitary gland, bone marrow and liver (Chen et al., 1996; Chen et al., 1997; Snow et al., 1998a). It has been demonstrated that the RGS16 protein not only regulates GPCR through classical signaling pathways, but also regulates tumor and inflammatory diseases through mitogen-activated protein kinase (MAPK), phosphoinositide 3-kinase (PI3K)/protein kinase B (Akt), Ras homolog family member A (Rho A) , stromal cell-derived factor 1 (SDF-1)/C-X-C motif chemokine receptor 4(CXCR4) and other signaling pathways (Johnson et al., 2003; Berthebaud et al., 2005; Liang et al., 2009). The association of the RGS16 protein with immune, inflammatory, tumor and metabolic disorders has been well established, and the RGS16 protein may also be involved in hepatitis B -induced inflammatory response. Therefore, the present review summarizes the structure, characteristics, regulatory mechanisms and known functions of RGS16 in different diseases such as immunity, inflammation, tumors and metabolic disorders.

## RGS protein family

**FIGURE 1:**
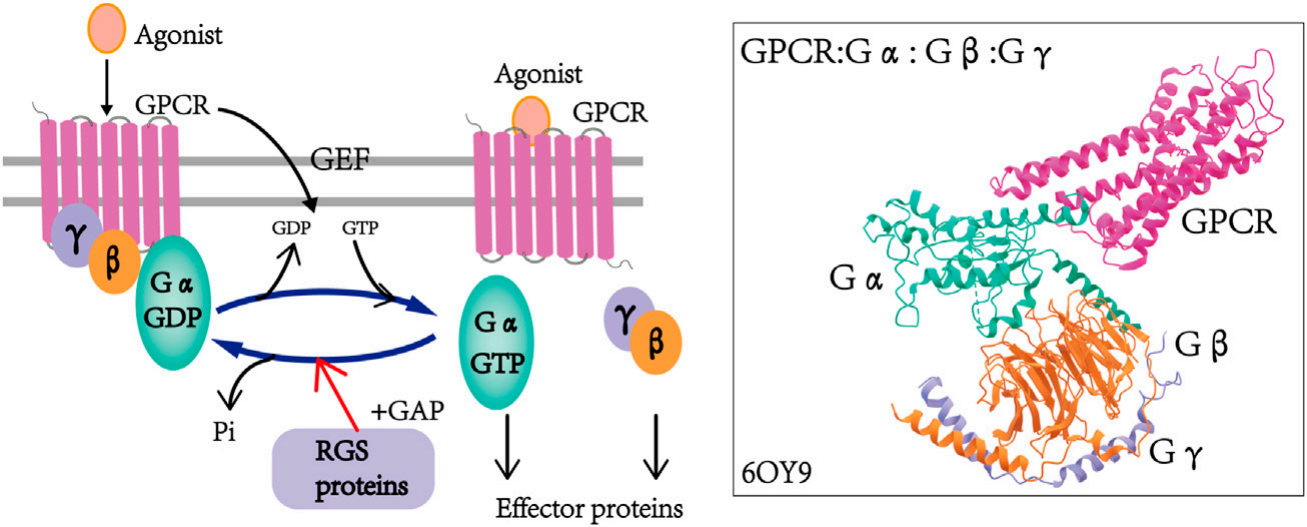
Regulation of GPCR by RGS proteins and the cycle of G protein activation and inactivation. In the presence of agonists, the GPCR undergoes conformational changes that induce the exchange of GDP on Gα to GTP and dissociation from Gβγ dimers, and in turn, the activated GTP-Gα and Gβγ dimers regulate downstream effectors. RGS proteins function as GAPs by accelerating the hydrolysis of GTP to GDP, thereby inactivating G proteins and finally leading to the termination of GPCR signaling. The structure of the G retinoid transduction complex was adopted from the Protein Data Bank: 6OY9 (GPCR: Gα: Gβ: G: γ) (Gao et al., 2019; Masuho et al., 2020).

At the end of the 20th century, a family of G protein signaling regulatory proteins (RGS) was identified in yeast, *C. elegans* and mammals (Chan and Otte, 1982; Dohlman et al., 1996; Koelle and Horvitz, 1996), and these represent a family of intracellular proteins of different molecular sizes, structures and multifunctionality that negatively regulate the signaling of GPCRs and heterotrimeric G proteins in a canonical manner (Koelle, 1997). RGS proteins control the strength and duration of the G protein-mediated signaling pathway, and they act as GTPase activating proteins (GAPs) which can accelerate the hydrolysis of the active Gα-GTP form of GTP, and then convert it into the inactive Gα-GTP form. This leads to the termination of the downstream G protein signaling pathway (Figure 1) (Gao et al., 2019; Masuho et al., 2020).

There are >30 RGS proteins in mammalian cells, all belonging to one superfamily, and all members have a shared, homologous, and highly conserved RGS domain or “RGS box” consisting of 120 amino acid residues (Almutairi et al., 2020). The RGS structural domain or “RGS box” mainly functions as GAPs (Koelle, 1997). However, it has also been reported that deletion of the RGS region resulted in the loss of GAP activity, while the binding of the Gα subunit was structurally unchanged (Ross and Wilkie, 2000; Sjögren et al., 2010; Kimple et al., 2011). Notably, there are different sequences on either side of the conserved RGS region, and this difference in sequence of the flanking regions may be a determinant of the functional specificity of RGS proteins. For instance, the RGS12 and RGS14 proteins share the RBD structural domain and Go Loco motif, through which both RGS12 and RGS14 interact with activated small G proteins, such as H-Ras-GTP and Rap-2-GTP (Willard et al., 2009; Shu et al., 2010). Unlike RGS14, RGS12 also possesses two additional structural domains including the PDZ and PTB domains. The C-terminus of C-X-C motif chemokine receptor 2 is bound by the PDZ domain (Snow et al., 1998b), while neuronal N-type calcium channels are involved in PTB domain interactions (Schiff et al., 2000; Richman et al., 2005). At the same time, both PDZ/PTB domains markedly attenuate extracellular regulated protein kinases (ERK) phosphorylation downstream of platelet derived growth factor-βreceptor (Sambi et al., 2006). This indicates that the sequence of the flanking region of the RGS proteins does serve an indispensable role in their function.

**FIGURE 2:**
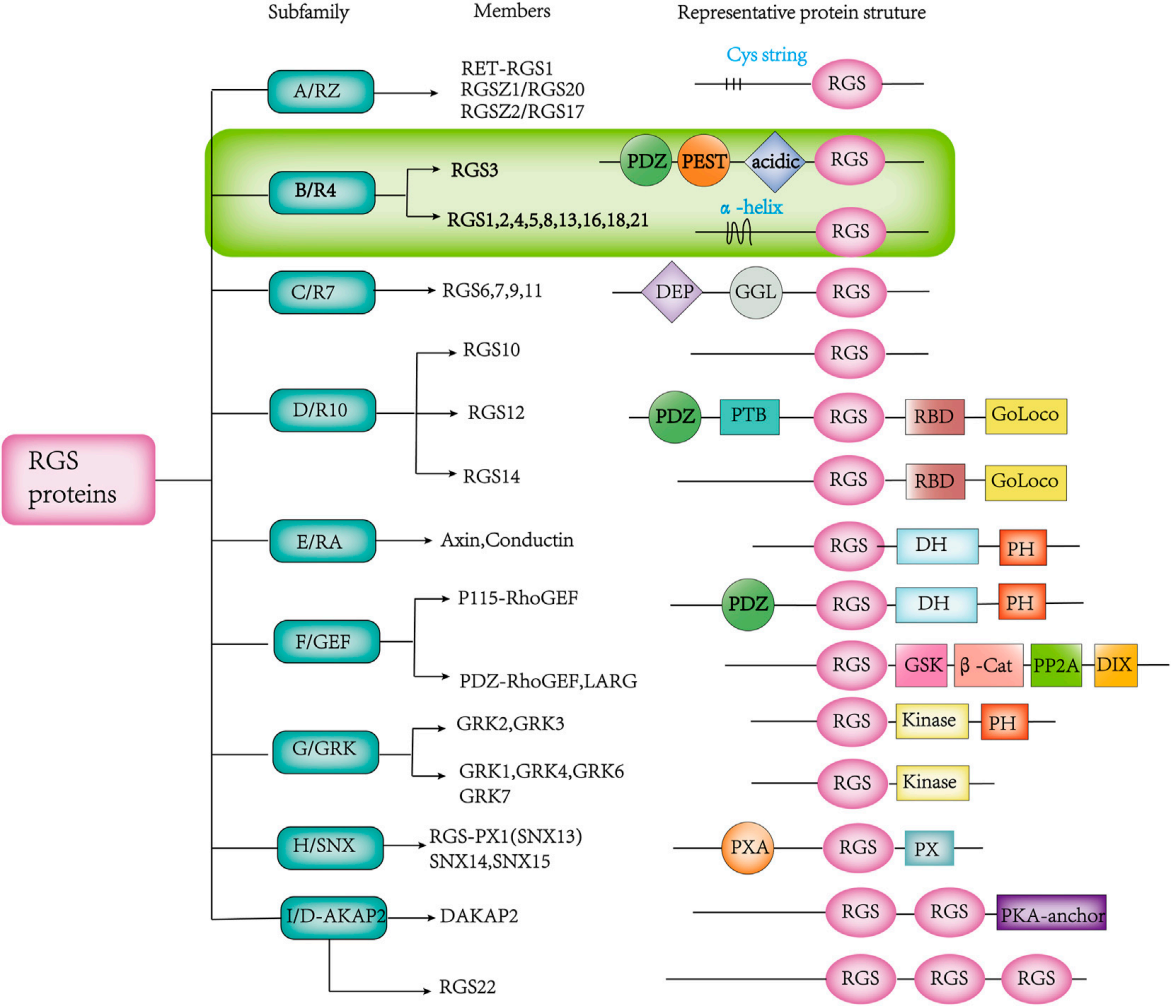
Various RGS protein subfamilies, along with their known members and distinguishing structures. The abbreviated representation of protein structural domain domains and patterns is as follows:β-Cat, β-catenin-binding; D-AKAP, dual-specificity A-kinase anchoring protein; DEP, disheveled/EGL-10/pleckstrin; DH, Dbl homology; DIX, disheveled homology domain; GAIP, G α interacting protein; GEF, guanine nucleotide exchange factor; GGL, G γ-like; GoLoco, Gα_i/o_-Loco; GRK, GPCR kinase; GSK, glycogen synthase kinase 3β-binding; PDZ, PSD95/D1g/Z0-1/2; PEST, proline, glutamine, serine, threonine-rich; PH, pleckstrin homology; PP2A, protein phosphatase 2A; PTB, phosphotyrosine binding; PX, phosphatidylinositol-binding; PXA, PX-associated; RBD, Ras-binding domain; RGS, Regulator of G protein Signaling domain; SNX, sorting nexin.

In recent years, more and more RGS proteins have been identified as research progresses. Based on the homology of amino acids sequences and the presence of external signaling domains, RGS proteins can be further divided into different subfamilies (Xie et al., 2016) (Figure 2). The RGS subfamily binds to different cognate Gα substrates through a unique stereochemical structure. The RGS subfamilies B/R4, C/R7 and D/R10 are involved in Gα _i/o_ (Berman et al., 1996; Snow et al., 1998b; Hooks et al., 2003; Bansal et al., 2007), the B/R4 subfamily is also involved in Gα_q_ (Heximer et al., 1997; Tany et al., 2022), the F/GEF subfamily is involved in Gα_12/13_ (Kozasa et al., 1998; Siehler, 2009), and the A/RZ subfamily is involved in Gα_z_ and Gα_i_ (Wang et al., 1998; Wang et al., 2002). The B/R4 subfamily is the largest subfamily in the RGS protein classification, but they are the smallest RGS proteins in terms of size (Bansal et al., 2007). This family is also the simplest of all RGS proteins in terms of structure and function. Unlike the B/R4 family, other families have multiple domains that interact with multiple proteins besides the Gα subunit and have more complex cellular effects (Almutairi et al., 2020).

Heterotrimeric G proteins catalyze the exchange of GTP on Gα with GDP upon conformational changes in GPCR, and the dissociation of activated GTP-Gα from Gβγ dimers to regulate downstream effector proteins that in turn generate a number of cellular responses, including cell proliferation, cell differentiation, plasma membrane transport, cell motility, and embryonic development (De Vries and Gist Farquhar, 1999; Wang et al., 2020). In this process RGS proteins control the strength and duration of G protein-mediated signaling pathways, leading to the termination of downstream signaling pathways, thus affecting these cellular processes (Masuho et al., 2020; O'Brien et al., 2019). They are involved in almost every aspect of cell biology and cause reactions in every organ system involved in disease processes such as cancer (Liang et al., 2009; Huang et al., 2020), inflammation (Suurväli et al., 2015; Xie et al., 2016), cardiovascular processes (Hendriks-Balk et al., 2008; Hayasaka et al., 2017), immunity and even depression (Muma, 2012). For instance, RGS1 is highly expressed in multiple myeloma and is a promising target for the treatment of multiple myeloma as a prognostic marker by desensitizing or stimulating receptor activity, thereby altering the GPCR signaling pathway and its downstream activity (Roh et al., 2017). Absence of RGS5 enhances angiotensin Ⅱ-induced blood pressure elevation and vasoconstriction, thereby preventing anxiety-like behavior and angiotensin Ⅱ-induced depression-like behavior (DʼSouza et al., 2019).

The classical biological role of RGS proteins at the cellular level is acting as GAPs to regulate the GPCR signaling pathway (Koelle, 1997). For example, RGS18, a myeloid-specific regulator of G protein signaling molecules highly expressed in megakaryocytes, acts as a GAP to regulate megakaryocyte differentiation and chemotaxis in mammalian and yeast cells *in vitro* via the GPCR signaling pathway (Yowe et al., 2001). In addition, RGS2, RGS4 and RGS5 act as GAPs attenuating the G protein signaling pathway in vascular and cardiac myocytes as well as in cells of the kidney and autonomic nervous system, which may be a possible strategy for the treatment and prevention of hypertension and cardiovascular disease (Gu et al., 2009). Nevertheless, in addition to acting as GAPs, RGS proteins are also regulated through non-classical pathways. For example, RGS5 reduces the proliferation of human ovarian cancer-derived primary endothelial cells via the MAPK/ERK signaling pathway under hypoxic conditions (Wang et al., 2019). RGS10, the most highly expressed protein in peripheral macrophages, inhibits the expression of cyclooxygenase-2 and tumor necrosis factor α (TNFα) through a G protein non-dependent mechanism to regulate inflammatory signaling in microglia and ovarian cancer cells (Alqinyah et al., 2018). RGS1 gene silencing inhibits the inflammatory response and angiogenesis in rheumatoid arthritis rats by suppressing the toll-like receptor (TLR) 3 signaling pathway (Hu et al., 2019). Overall, the RGS protein family serves a critical role in the regulation of G protein-mediated pathways and many pathophysiological processes in various tissues through both classical and non-classical pathways. Synchronously, the RGS16 protein has been predicted to serve a central role in immune and inflammatory responses in addition to being a key member of the RGS family of oncogenes that contribute to the malignant progression of a number of human cancer types, but this remains to be further investigated (Xie et al., 2016).

## Characteristics and functions of RGS16

### Orientation and structure

**FIGURE 3:**
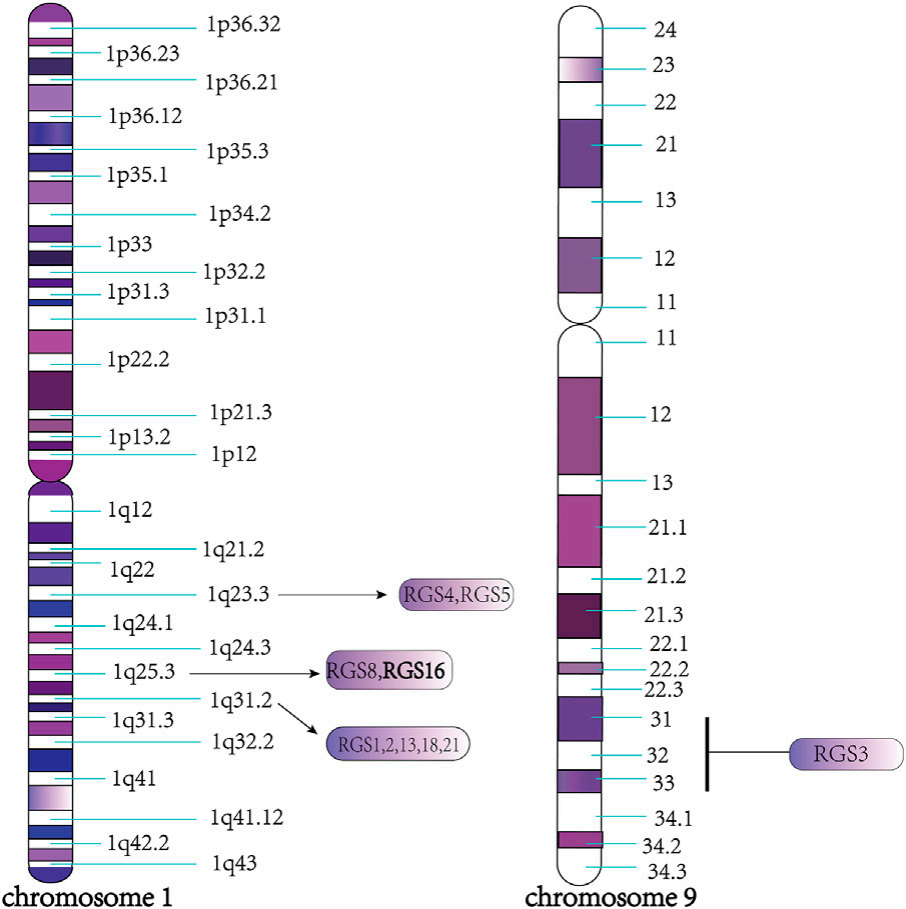
Specific region of each protein chromosome of B/R4 subfamily. As shown in the figure, all the members of the B/R4 subfamily are located on chromosome 1, except RGS3, which is located on chromosome 9, such as RGS4 and RGS5 on 1q23.3, RGS8 and RGS16 on 1q25.3 RGS1, 2, 13, 18, 21 on 1q31.2.

The RGS16 protein belongs to the B/R4 subfamily of RGS proteins, which are highly conserved in mammals. In humans and mice, most of the genes encoding B/R4 subfamily proteins are located on chromosome 1, except for RGS3, which is located on chromosome 9, and composed of two or more clusters of genes, such as RGS4 and RGS5 on 1q23.3, RGS8 and RGS16 on 1q25.3, and RGS1, 2, 13, 18 and RGS21 on 1q31.2 (Sierra et al., 2002) (Figure 3). Furthermore, the RGS1/RGS16 neighboring region constitutes a synteny group that is highly conserved in tetrapods, and genes in this region are closely homologous to major histocompatibility complex (MHC) or other MHC paralogs on chromosome 6, providing a useful marker for studying the origin and evolution of MHC (Suurväli et al., 2013).

**FIGURE 4:**
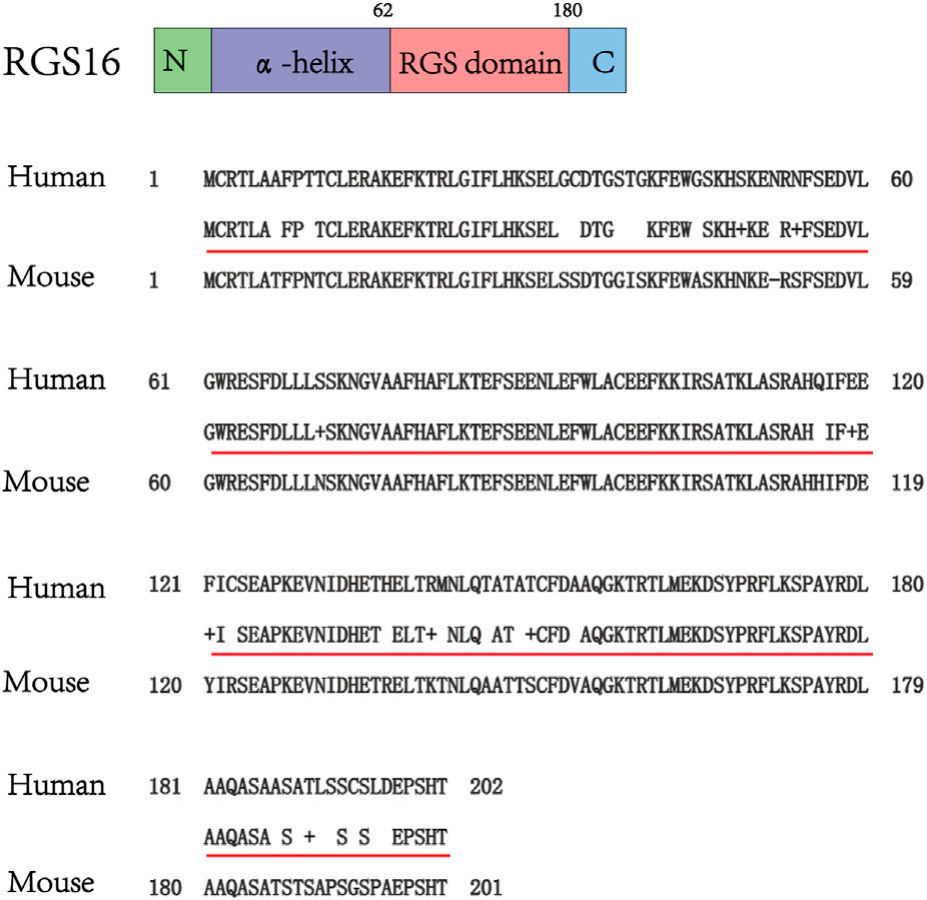
Schematic structure of RGS16, amino acid sequence homology analysis of human and mouse RGS16 (data from GenBank). Query is human, Sbjct is mouse, the middle row indicates where the human and mouse sequences are identical, and the other blanks or + signs are where the sequences are different.

**FIGURE 5:**
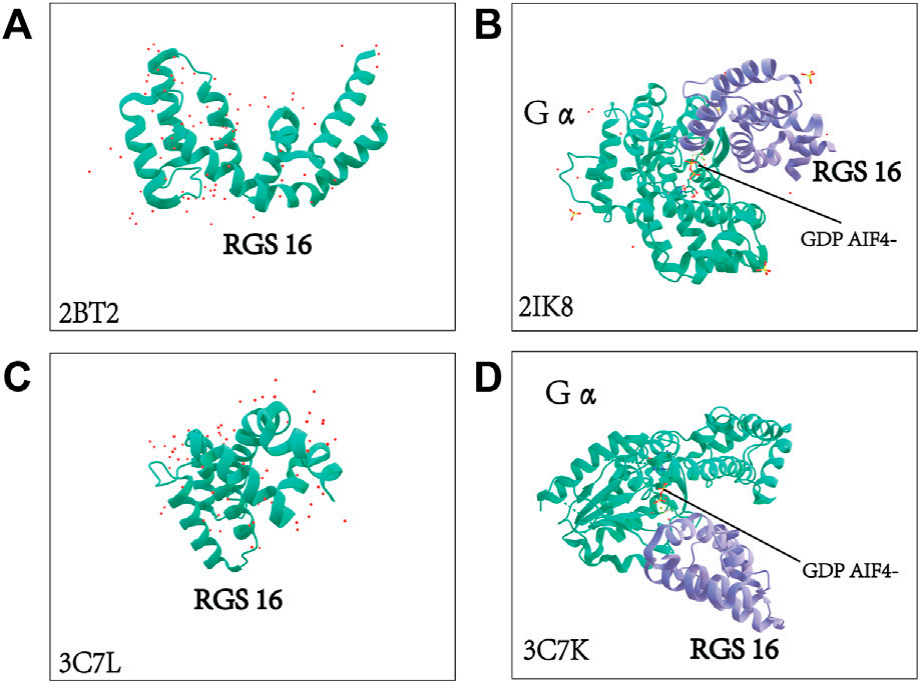
Crystal structure of RGS16 and crystal structure of Gα complexed with RGS16. The molecular structure of Gα and the structural basis for RGS16-mediated inactivation were adopted from the Protein Data Bank. 2BT2: crystal structure of Homo sapiens-derived RGS16 2IK8: Human sapiens-derived crystal structure of Gα in complex with RGS16 3C7L: crystal structure of Mus musculus-derived RGS16 3C7K: Mus musculus-derived crystal structure of Gα in complex with RGS16 (Slep et al., 2008; Soundararajan et al., 2008).

RGS16 was originally cloned from the retina by Snow BE et al. At the same time, they revealed that RGS16 is highly expressed in the retina and is also involved in the visual signaling pathway (Chen et al., 1997; Snow et al., 1998a; Faurobert et al., 1999). With the exception of the RGS3 protein, other members of the B/R4 subfamily, including RGS16, also contain short, disordered amino- and carboxy-terminal extensions and an α-helix based on a conserved and functional RGS domain (Bansal et al., 2007). RGS16 contains a core RGS structural domain, located between amino acids 62 and 180. This structural domain is highly conserved between yeast and mammalian RGS proteins (Faurobert and Hurley, 1997). Human RGS16 is most similar to RGS3 (175 amino acids at the C-terminus, GenBank U27655), and mouse RGS16 (U72881) shares 86% identity with human (Chen et al., 1996; Snow et al., 1998a). (Figure 4)A very short unique membrane binding domain with amphiphilic characteristics at the NH2 terminus of RGS16 provides the structural basis for its membrane binding and biological activity (Chen et al., 1999). The core RGS domain of RGS16 maintains full G-protein binding and GTP-activating protein activity *in vitro*, but its NH2-terminal domain is also required for *in vivo* membrane binding and bioactivity, as evidenced by its ability to attenuate pheromone signaling in yeast (Chen et al., 1999). Membrane interacting protein of RGS16 (miR16), a putative membrane glycerol phosphodiester phosphodiesterase, is required for its interaction with the RGS structural domain of RGS16, and analysis of deletion mutants suggests that the N-terminal region of the RGS domain in RGS16 is required for its interaction with miR16 (Zheng et al., 2000). Novel interactions between RGS proteins (RGS4, RGS5and RGS16) and the multifunctional protein 14-3-3 have been identified, and their interactions do not depend on any post-translational modifications, with the 14-3-3 protein acting as a molecular chelator that selectively blocks RGS proteins from interacting with Gα and ultimately prolongs or enhances specific G protein-mediated signaling (Abramow-Newerly et al., 2006). A comprehensive understanding of the structure of the RGS16 protein and its interaction is crucial, and the crystal structure of Gα in complex with RGS16 in the GTP hydrolysis transition state is shown in Figure 5 (Slep et al., 2008; Soundararajan et al., 2008).

### Regulation of RGS16 expression and function

RGS16is also known as A28-RGS14 or RGS-rand is expressed in a variety of tissues and cells, being expressed at high levels in the retina, pituitary gland, bone marrow and liver (Chen et al., 1996; Chen et al., 1997; Snow et al., 1998a). It has emerged as an important regulator of cellular processes under different pathological and physiological conditions. Examples of RGS16 protein, that function as GAPs at the cellular level is regulation of the intensity and duration of the entire G protein signaling process, thereby terminating the G protein signaling pathway, are as follows: RGS16 interacts with protease-activated receptor 2 (PAR2) in the presence of Gα_i_ to inhibit PAR2/Gα_i_-mediated signal transduction (Kim et al., 2018). RGS16 binds to the β2 adrenergic receptor and suppressed Gα_S_ mediated signal transduction. The role of RGS16 in regulating the response of osteoblastic ovarian cancer G protein-coupled receptor 1 to metabolic acidity stimulates osteoclast bone resorption capacity, as GAPs affect downstream signaling, which contributes to the understanding of bone loss in patients with chronic nephropathy acidosis (Krieger and Bushinsky, 2021). In summary, RGS16 protein acts through the classical GPCR pathway, while subsequent studies have illustrated that RGS16 protein also influences disease processes through non-classical pathways, including the MAPK, PI3K/Akt, Rho AandSDF-1/CXCR4 pathways (Johnson et al., 2003; Berthebaud et al., 2005; Liang et al., 2009). Liang et al. revealed that RGS16 protein expression is upregulated in breast cancer cells and inhibits the activation of the PI3K signaling pathway via epidermal growth factor receptor (EGFR), thereby suppressing the proliferation of breast cancer cells (Liang et al., 2009).

Studies have demonstrated that the activity and function of RGS16 protein can be affected by post-translational modifications, including phosphorylation and palmitoylation (Chen et al., 1999; Druey et al., 1999; Chen et al., 2001; Cunningham et al., 2001). Data have indicated that the RGS16 protein contains two conserved tyrosine residues, Tyr168 and Tyr177, in the RGS box, which are predicted phosphorylation sites bi-directionally regulating the GAP activity of RGS16 (Chen et al., 2001). EGFR-mediated tyrosine phosphorylation of the RGS16 protein at the Tyr168 residue reduces its GAP function on the Gα subunit, thereby impairing its inhibition of ligand-stimulated MAPK activity. Mutation of the Tyr177 residue of RGS16 does not affect its GAP activity *in vitro*, but eliminates the ability of RGS16 to regulate Gq-coupled MAP kinase activation or Gi-mediated inhibition of adenylate cyclase (Derrien and Druey, 2001). Instead of affecting the intracellular localization or GAP activity of RGS16, Alexandrine Derrien et al. verified that tyrosine kinase-mediated phosphorylation of RGS16 did not affect its intracellular localization, but promoted its stability (Derrien et al., 2003). Additionally, RGS16 is constitutively phosphorylated at the serine 194 site when expressed in HEK293T cells, whereas in cells expressing the α2A adrenergic receptor, serine 53 is phosphorylated in a ligand-dependent manner in response to adrenergic stimulation. Phosphorylation of these two sites impairs their GAP activity and subsequently attenuates heterotrimeric G protein-stimulated extracellular signal-regulated protein kinase activity (Chen et al., 2001). S-palmitoylation, or S-acylation, refers to the covalent modification of long-chain fatty acids (usually 16-carbon palmitic acid) to protein cysteine residues via thioester bonds, which occurs on several molecules of the G-protein linkage signaling pathway, and is a dynamic and reversible form of post-translational modification that is widely present in living organisms and serves an important role in regulating protein transport, cellular localization and stability, and is involved in a number of biological processes and closely related to the occurrence and development of numerous diseases (Druey et al., 1999). In a previous study, RGS16 was involved in the membrane localization at the NH2-terminal palmitoylation sites Cys-2 and Cys-12 of the RGS box (De Vries et al., 1996; Druey et al., 1999). Furthermore, Chen C et al. reported palmitoylation of RGS16 in *Saccharomyces cerevisiae*, but this modification does not require plasma membrane binding or adapter signaling regulation (Chen and Lin, 1998; Chen et al., 1999). Overall, there may be differences in palmitoylation of RGS16 among different species; however, this usually has an effect on the activity of RGS16.

### Role of RGS16 in tumors

RGS proteins and GPCR-mediated signaling pathways often serve a key role in tumorigenesis, and certain hallmark oncogenic processes, such as uncontrolled growth, invasion, and metastasis, can be attributed to alterations in GPCR signaling pathways. Therefore, as a member of the RGS protein family and an important regulatory protein of the GPCR pathway, RGS16 undoubtedly serves a nonnegligible role in tumorigenesis. More specifically, recent studies have found that the RGS16 protein is associated with a variety of cancer types, including breast cancer (Wiechec et al., 2008; Liang et al., 2009; Wiechec et al., 2011; Hoshi et al., 2016), pancreatic cancer (Kim et al., 2010; Carper et al., 2014; Ocal et al., 2015; Zolghadri et al., 2018; Layeghi-Ghalehsoukhteh et al., 2020), colorectal cancer (Buckbinder et al., 1997; Miyoshi et al., 2009), neuroblastoma (Liu et al., 2005; Airoldi et al., 2006), glioma (Huang et al., 2020), chondrosarcoma (Sun et al., 2015), hyper diploid acute lymphoblastic leukemia in children (Davidsson et al., 2007) and other malignant tumors. RGS16 has been studied in breast cancer. Allelic imbalance mapping analysis verified that the 1q25.3 region where the RGS16 protein is located is highly unstable in breast tumors, and RGS16 expression was reduced in breast cancer samples due to allelic imbalance, intragenic chromosomal break points and methylation (Wiechec et al., 2008). Additionally, δEF1 family proteins (δEF1/zinc finger E-box binding homeobox 1 and Smad interacting protein-1/zinc finger E-box binding homeobox 2), key regulators of epithelial-mesenchymal transition (EMT), inhibit RGS16 expression and promotebreast cancer cell invasion, suggesting that low RGS16 expression may contribute to the promotion of cancer cell motility by δEF1 family proteins (Hoshi et al., 2016). On the contrary, high RGS16 expression in breast tumors attenuates the growth factor-induced PI3K signaling pathway, thereby inhibiting proliferation, human epidermal growth factor receptor 2 activation and resistance to chemotherapeutic agents in breast tumor cells (Liang et al., 2009). Overall, these discoveries indicate that RGS16 has a critical effect in breast cancer progression through allelic analysis, δEF1 family protein inhibition and GPCR-independent pathways, demonstrating that RGS16 may be a novel therapeutic target in breast cancer.

In parallel to the investigation of RGS16 in breast cancer, several relevant studies have also linked changes in RGS16 expression to poor prognosis of cancer. For example, in patients with pancreatic cancer with lymph node metastasis, the RGS16 and FosB expression is markedly reduced in pancreatic cancer andis closely associated with a decreased survival rate of patients (Kim et al., 2010). Expression of RGS16- green fluorescent protein (GFP), which inhibits Gi/q-coupled GPCRs, negatively regulates pancreatic ductal adenocarcinoma (PDA) progression and can be used for rapid preclinical *in vivo* validation of novel chemotherapeutic agents targeting early lesions in patients at high risk for successful resection or progression to PDA. RGS16-GFP has recently been reported in caerulein-induced acinar cell dedifferentiation, early tumor and throughout PDA progression (Layeghi-Ghalehsoukhteh et al., 2020) (Ocal et al., 2015). This suggests that RGS16 may be used as a prognostic marker for pancreatic cancer and PDA. Additionally, a recent study has suggested that RGS16 can be used as a diagnostic biomarker for immune subtypes of ovarian cancer and as a biomarker to predict the clinical stage of the disease (Hu et al., 2021).

In colorectal cancer, however, RGS16has a completely different biological function. RGS16 mRNA and protein expression in colorectal cancer tissues is higher than that in normal tissues, but the prognosis of patients with high RGS16 expression is worse than that of patients with low RGS16 expression, and RGS16 can be used as a prognostic indicator for patients with colorectal cancer (Miyoshi et al., 2009). Other recent *in vitro* experiments have revealed that RGS16 is oversaturated in glioma cell lines and facilitates tumor cell proliferation and migration through the EMT process, suggesting the potential of RGS16 as a novel prognostic biomarker and therapeutic target (Huang et al., 2020). Therefore, such studies imply that RGS16 may serve as a biomarker for diagnosis and prognosis in cancer. Many studies (summarized in Table 1) have shown that RGS16 expression is regulated by various stimuli in different cell types and disease models.

**TABLE 1:** Pathophysiological roles of RGS16.

Samples sources	Stimuli/Disease model	mRNA/Protein	Expression	Year	References
Primary bone cell	Chronic metabolic acidosis (MET)	mRNA/Protein	Decrease	2021	Krieger and Bushinsky, (2021)
Tumor and blood DNA samples	Primary sporadic breast cancer	mRNA/Protein	Decrease	2008	Wiechec et al. (2008)
Breast cancer cells	dEF1 family proteins	mRNA/Protein	Decrease	2015	Hoshi et al. (2016)
Breast cancer cell line MCF7	EGF/PI3K	mRNA/Protein	Decrease	2009	Liang et al. (2009)
Pancreatic cancer tissue specimens	Pancreatic cancer with lymph node metastasis	mRNA/Protein	Decrease	2010	Kim et al. (2010)
KIC; RGS16::GFP mice	Pancreatic ductal adenocarcinoma (PDA)	mRNA	Increase	2020	Layeghi-Ghalehsoukhteh et al. (2020)
KIC; RGS16::GFP mice	Pancreatic ductal adenocarcinoma (PDA)	mRNA	Increase	2015	Ocal et al. (2015)
Twenty-two cell lines derived from human gastrointestinal cancer	Gastrointestinal cancer	mRNA/Protein	Increase	2009	Miyoshi et al. (2009)
Human neuroblastoma BE (2)-C and SH-SY5Y cell lines	Retinoic acid-induced neuroblastoma cells	Protein	Decrease	2005	Liu et al. (2005)
The CGGA microarray database	Glioma	mRNA	Increase	2020	Huang et al. (2020)
Primary Human Chondrosarcoma Tissue; Chondrosarcoma cell line JJ	MIR-181a/Chondrosarcoma	mRNA/Protein	Decrease	2015	Sun et al. (2015)
The array CGH study	Hyper diploid acute lymphoblastic leukemia	mRNA	Increase	2007	Davidsson et al. (2007)
The UCSC Xena database	Ovarian cancer	mRNA	Increase	2021	Hu et al. (2021)
THP1	LPS(1 ug/ml) Pam3CysSK4(10 ng/ml)2, 4, 6, 8 and24 h	mRNA	Increase	2015	Suurväli et al. (2015)
DC	LPS (10 ng/ml) IL-10 (50 ng/ml)2 and 8 h	mRNA	Increase	2004	Shi et al. (2004)
PBCs; U937 and the 293 human embryonic kidney cell lines	IL-2 (500 Pm)	mRNA/Protein	Increase	1999	Beadling et al. (1999)
RGS16^−/−^ mice; Th1, Th2, or Th17	Pulmonary inflammation	mRNA/Protein	Increase	2012	Shankar et al. (2012)
RGS16 Tg mice	Allergic inflammation	mRNA	Increase	2003	Lippert et al. (2003)
B cell; 70Z/3 cell line	IL-17 (30 ng/ml) 5,15,30,60 min	mRNA/Protein	Increase	2010	Xie et al. (2010)
CD4+Tcell	IL-17 (30 ng/ml) 1, 4 and 24 h	mRNA/Protein	Increase	2013	Ding et al. (2013)
CD8^+^ splenic T cells from the RGS16mCherry-Cre-ERT2	Promotes antitumor CD8^+^ T cell exhaustion	mRNA/Protein	Increase	2022	Weisshaar et al. (2022)
Porcine kidney cell line PK-14/A	LPS(2.5 ug/ml) PHA (1 ug/ml) ConA(5 ug/ml) polyI:C (5 ug/ml)	mRNA	Increase	2009	Timmusk et al. (2009)
Porcine kidney cell line PK-15/A	Porcine circovirus type 2 (PCV2)	Protein	Decrease	2015	Choi et al. (2015)
C57BL/6 mice	During fasting	mRNA/Protein	Increase	2006	Huang et al. (2006)
INS-1-derived 832/13 rat insulinoma cells	Carbohydrate response element binding protein (ChREBP)	mRNA	Increase	2016	Sae-Lee et al. (2016)
AML12 cells; AAV8-Arg2 db/db mice	Non-alcoholic fatty liver disease	mRNA/Protein	Decrease	2019	Zhang et al. (2019)
Primary hepatocyte; HEK293A; Hepa1-6; C57BL/6J	Bioactive lipid accumulation, and hepatic inflammation	mRNA/Protein	Decrease	2021	Bai et al. (2021)
RGS16::GFP mice	Embryonic endocrine pancreas and mouse models of diabetes	Protein	Increase	2010	Villasenor et al. (2010)
Male Wistar rats and C57BL/6N mice	6-month-old Wistar rats infused with glucose or saline for 72 h; Isolated rat islets exposed to 2.8 or16.7 mM glucose for 24 h	mRNA	Increase	2016	Vivot et al. (2016)
MO7e cells	During megakaryocyte differentiation	mRNA	Increase	2006	Kim et al. (2006)

In terms of malignancies, synergistic expression of dual specificity phosphatase 6 and RGS16 blocks the growth of retinoic acid-induced neuroblastoma cells (Liu et al., 2005). A novel mechanism by which microRNA-181a increases CXCR4 signaling through inhibition of RGS16 protein promotes chondrosarcoma growth, angiogenesis, and metastasis (Sun et al., 2015). Finally, RGS16 protein is aberrantly expressed in hyper diploid acute lymphoblastic leukemia, suggesting that RGS16 may be involved in hematologic malignancies (Davidsson et al., 2007). Above all, RGS16 protein exerts a critically influential regulatory effect in these malignancies through GPCRs and other non-classical signaling pathways, and has the to be a potential therapeutic target for the regulation of tumor processes.

### RGS16 in immunity and inflammation

Immune cells are involved in the development of numerous diseases, and a number of key regulatory molecules can participate in the progression of related diseases by affecting the function of these immune cells. Similar to our and other teams’ previous studies, hepatitis B e antigen (HBeAg) induces activation of macrophages via the TLR-2/nuclear factor-κB (NF-κB) signaling pathway, further aggravating liver fibrosis (Xie et al., 2021) and the ERK/cyclic adenosine monophosphate (cAMP)-response element binding protein/microRNA-212–3p negative feedback loop to inhibit HBeAg-induced macrophage activation, and thus, aggravates liver injury (Chen et al., 2020). As one of the newly discovered molecules, accumulating data have indicated that RGS16 is expressed in various immune cells such as monocytes (Suurväli et al., 2015), T lymphocytes (Beadling et al., 1999), dendritic cells (Shi et al., 2004) and natural killer cells (Kveberg et al., 2005). Therefore, this section mainly summarizes the expression of RGS16 in a variety of immune cells and its known functions in inflammation.

RGS16 is a crucial modulator of inflammatory responses and can inhibit pro-inflammatory responses (Shankar et al., 2012). Suurväli J reported that overexpression of RGS16 inTHP-1 cells was associated with decreased production of the pro-inflammatory cytokines interleukin (IL)-1β, IL-6 and TNFα after lipopolysaccharide (LPS) stimulation, while RNAi knockdown of RGS16 in THP-1 cells was associated with increased expression of the pro-inflammatory cytokines IL-1β, IL-6 and TNFα after LPS stimulation (Suurväli et al., 2015). Furthermore, the RGS16 gene was up-regulated 100 times in human monocyte-derived dendritic cells treated with LPS compared with untreated cells (Perrier et al., 2004). One of the mechanisms by which TLRs, important pattern recognition receptors on dendritic cells, alter GPCR signaling is by altering RGS expression, and it has been demonstrated that TLR3 or TLR4 is involved in the induction of RGS16 expression on monocyte-derived dendritic cells in human and mice, although more so in human cells than in mouse cells (Shi et al., 2004). Therefore, RGS16 expression was markedly increased on monocytes and monocyte-derived dendritic cells after LPS treatment, implying that RGS16 may be engaged in innate immune-related inflammatory diseases.

In addition, RGS16 expression is markedly altered in B and T cells when they are exposed to different conditions, suggesting that RGS16 also serves an essential role in adaptive immunity (Estes et al., 2004). It has been demonstrated that RGS16 expression is upregulated in human primary T lymphocytes after IL-2 stimulation; however, excessive cAMP inhibits RGS16 expression on T cells (Beadling et al., 1999). Allergic respiratory inflammation often accompanies human asthma and produces inflammatory responses such as allergy cytokine (IL-3, IL-4, IL-5) production, airway eosinophil recruitment, presence of immunoglobulin E (atopic) and increased mucus secretion from cupped cells. However, the accumulation of these inflammatory changes can lead to airway sensitivity to narrowing triggers, causing allergic airway inflammation which has been reported to be associated with RGS16 (Lambrecht and Hammad, 2015). Chemokine-induced recruitment of T lymphocytes to the lung is essential for allergic inflammation, and RGS16 has been demonstrated to affect T cell migration and activation by restricting the signaling pathways of chemokine receptors CXCR4, C-C motif chemokine receptor 3 and C-C motif chemokine receptor 5, thereby affecting allergic inflammation (Lippert et al., 2003; Shankar et al., 2012). In earlier studies, RGS16 transgenic mice in an allergic airway model had reduced numbers of lung T helper 2 (Th2) cells and markedly increased numbers of Th2 cells produced by lymphoid-like organs, leading to severe generalized inflammation and airway hyperresponsiveness (AHR) disease (Lippert et al., 2003). There are also reports that RGS16 suppresses pulmonary inflammation by regulating chemokines, such as C-C motif chemokine receptor 4, C-C motif chemokine receptor 10, and C-C motif chemokine ligand 17, which mediate T-cell restriction of Schistosoma mansoni granulomas (Shankar et al., 2012). RGS16 protein has also been reported to attenuate the lung T helper 17 cell inflammatory response (Shankar et al., 2012). Recent literature indicates that RGS16 synergizes with programmed cell death protein 1 blockade and promotes antitumor cluster of differentiation 8-positive T cell failure in an ERK1-dependent manner (Weisshaar et al., 2022). Similar to T cells, the function of B cells, one of the important adaptive immune cells, is also regulated by RGS16. The germinal center (GC) is a structure located within the secondary lymphoid follicles and is the site of proliferation, selection, maturation and death of B cells (Mesin et al., 2016). Spontaneous GC formation exists in mouse models of autoimmune disease, and human autoimmune disease may be associated with GC formation or dysfunction (Linterman et al., 2009; Craft, 2012). Specific cellular foci that promote the interactions of antigen presentation and T and/or B cells during normal immune responses are referred to as spontaneous GCs. These are formed in the presence of C-X-C motif chemokine ligand 12(CXCL12) and C-X-C motif chemokine ligand 13, while RGS proteins downregulate lymphocyte responses to these chemokines, thereby retarding B and T cell migration (Walker et al., 2003). It has been reported that RGS16 is mainly expressed in cluster of differentiation 4-positive (CD4^+^) T cells and B cells of GCs, which can accelerate the intrinsic rate of the Gα GTPase reaction and down-regulate the response of lymphocytes to chemokines, thus delaying the migration of CD4^+^ T cells and B cells of GCs (Lippert et al., 2003) (Ding et al., 2013). For example, IL-17 targets self-reactive B cells in BXD2 mice, rapidly activates the NF-κB signaling pathway, leads to upregulation of RGS16 expression in spontaneous development centers, delays B cell migration, and promotes the formation of spontaneous GCs (Xie et al., 2010) (Hsu et al., 2008). These data all suggest that RGS16 serves an important role in the migration of T and B cells.

RGS16 is utilized not only by organisms to regulate the function of various immune cells, but also by pathogens to disrupt the host immune response and promote inflammatory responses. Sequence analysis of the non-structural protein encoded by porcine circovirus type 2 (PCV2) open reading frame 3 (ORF3) indicates that it is closely related to human and murine RGS16. Immunofluorescent labeling has confirmed the induced expression of poRGS16 at the protein level and revealed that PCV2 ORF3 protein co-localizes with poRGS16 in LPS-activated porcine peripheral blood mononuclear cells (Timmusk et al., 2009). Additionally, the PCV2 ORF3 protein promotes the degradation of RGS16, further enhances the nuclear translocation of NF-κB through the ERK1/2 signaling pathway, and promotes the secretion of IL-6 and IL-8 from porcine epithelial cells, which is the reason why a severe inflammatory response and leukocyte infiltration will be induced around the host cells early in PCV2 infection (Choi et al., 2015). This is a good example of how pathogens can utilize the RGS16 protein to disrupt the host immune response and promote inflammation. Recently Seung-Hoon Lee et al. detected single nucleotide polymorphisms (SNPs) in the RGS16 region, which were associated with PCV2 viral load in 22 identified SNPs and in both haplotype and double haplotype analyses, and in conclusion, they demonstrated that RGS16 SNPs affect PCV2 viral load (Lee et al., 2021).

### RGS16 in biorhythms and metabolism

As one of the candidate biological clock/bio-clock control genes, RGS16 is mainly expressed in the suprachiasmatic nucleus (SCN) and thalamus of the brain and in the liver, suggesting that RGS16 is associated with central and peripheral circadian clocks (Grafstein-Dunn et al., 2001; Ueda et al., 2002; Hayasaka et al., 2011). The SCN is the main circadian pacemaker in mammals and is a network structure consisting of multiple types of neurons. A key feature of RGS16 is that it functions in the SCN in a time-specific manner (Mieda et al., 2015). G protein-coupled receptor 176 (Gpr176) is an orphan SCN-rich GPCR that determines the speed of the central clock of the SCN (Doi et al., 2016). Naoto Hayasaka et al. demonstrated that RGS16 is involved in two independent but interacting circadian rhythm systems, light-entrainable oscillator and food-entrainable oscillator. Compared with wild-type mice, RGS16 knockout mice exhibited shorter rhythms of locomotor activity and reduced total activity. In addition, SCN-controlled food anticipatory activity was diminished when feeding was restricted during the day in these knockout mice, suggesting that RGS16 is closely associated with Gpr176 and regulates central biological rhythmic processes via the GPCR pathway (Hayasaka et al., 2011).

In terms of the peripheral biological clock, Huang J reported that RGS16 is predominantly expressed in periportal hepatocytes during the last hours of the daily fast, which are predominantly lipolytic and gluconeogenic (Huang et al., 2006). Interestingly, it has been previously demonstrated that RGS16 mRNA and protein are upregulated during fasting and rapidly downregulated upon resumption of fasting (Huang et al., 2006). Compared with wild-type mice, RGS16 knockout mice developed fatty liver after 10 days of a high-fat, high-carbohydrate diet and exhibited higher fatty acid oxidation rates and plasma β-ketone levels, in contrast to transgenic mice expressing RGS16 protein specifically in the liver, which exhibited the opposite phenotype and low glucose levels and developed fatty liver after overnight fasting. The glucose-dependent transcription factor carbohydrate response element-binding protein (ChREBP) induces fatty acid synthesis, which in turn is required to induce RGS16 expression during fasting, suggesting that RGS16 feedback inhibits fatty acid oxidation in the liver (Sae-Lee et al., 2016). Furthermore, Zhang Y et al. revealed that arginase 2 (Arg2) was a fasting-induced hepatocyte factor and was upregulated under fasting conditions and after treatment with alginate, an inhibitor of ChREBP, to prevent liver and peripheral fat accumulation, liver inflammatory response, insulin, and poor glucose tolerance in an obese mouse model. Interestingly, Arg2 can inhibit RGS16 expression and RGS16 gene recombination can reverse the effects of Arg2 overexpression, suggesting that Arg2 reduces hepatic fat accumulation in hepatocytes by inhibiting RGS16 (Zhang et al., 2019). Circadian-induced expression of G0/G1 switch 2 and RGS16 in hepatocytes, which regulates substrate oxidation in resting hepatocytes, has recently been reported to reduce liver inflammation and fibrosis in obese mice, suggesting that RGS16 is essential for maintaining liver health (Bai et al., 2021). Therefore, RGS16 expression in hepatocytes is controlled by feeding and fasting and can inhibit fatty acid metabolism through ChREBP and promote lipid synthesis under the control of Arg2. Apart from being influenced by biorhythms in the liver, RGS16 has also been reported to be influenced by biorhythms in the pancreas. Mice with deletion of the RGS16 gene develop a dedifferentiated exocrine pancratia at 2 months of age and become malnourished, underweight, hypoglycemicand hypothermic (Zolghadri et al., 2018). RGS16 and RGS8 are expressed in embryonic pancreatic progenitor and endocrine cells and disappear in adults; however they are reactivated in type I and II diabetes models, which suggests that the RGS16 and RGS8 proteins may become a novel target for further treatment of diabetes (Villasenor et al., 2010). In rodent and human islets, RGS16 is a novel regulator of β-cell function that promotes insulin secretion and β-cell proliferation by limiting the tonic inhibitory signal exerted on islets through δ-cell-derived somatostatin (Vivot et al., 2016). Overall, RGS16 may be an active therapeutic strategy for the treatment of fat accumulation, liver inflammatory diseases, and insulin and glucose intolerance mediated by biorhythms.

### Effect of RGS16 in coagulation

Platelets have no nucleus and are cytoplasmic fragments derived from bone marrow megakaryocytes (Rendu and Brohard-Bohn, 2001). Their principal function is to promote hemostasis and accelerate clotting by responding, together with clotting factors, to bleeding from vascular injury (Ross et al., 1988). Additionally, RGS16 protein is highly expressed in megakaryocytes and platelets (Berthebaud et al., 2005; Kim et al., 2006). It has recently been proposed that RGS16 moderate’s platelet function and thus influences the coagulation process (Zhang et al., 1999; Berthebaud et al., 2005; Karim et al., 2016; Hernandez et al., 2019). Previously, it has been observed that overexpression of RGS16 in the megakaryocyte MO7e cell line inhibitsSDF-1-induced migration and leads to MAPK and AKT inactivation, thereby affecting platelet coagulation (Berthebaud et al., 2005). The platelet-secreted chemokine CXCL12 activates platelets in an autocrine/paracrine manner (Walsh et al., 2015). Compared with wild-type mice, platelets in a RGS16 knockout mouse model exhibited increased protease-activated receptor 4 and collagen-induced aggregation, as well as markedly increased agonist-dependent platelet aggregation, dense granule and alpha granule release, integrin αIIbβ3 activation, and phosphatidylserine exposure after CXCL12 stimulation. In addition, ERK and Akt phosphorylation levels in platelets were also considerably enhanced after CXCL12 stimulation. These phenomena suggest that RGS16 serves an important role in the coagulation process by regulating platelet activation through CXCL12 (Karim et al., 2016). Similarly, the RGS16 protein attenuates the stimulation of MAPK p38 by G protein-coupled platelet-activated factor PAF receptor, potentially affecting the function of the PAF receptor in coagulation (Zhang et al., 1999). Based on the above, RGS16 protein may also affect platelet function through some non-classical pathways, which is not well studied, but may be a potential mechanism for the treatment of coagulation disorders.

## Conclusion and prospects

Since its discovery at the end of the 20th century, there has been tremendous progress in understanding the structure, function and regulatory mechanisms of RGS16, as well as its potential role in various pathophysiological states. Despite its simple structure, the studies discussed in the present review suggest that RGS16 is an important regulator in immune, inflammatory, tumor, biological rhythm and metabolic disorders, and coagulation dysfunction. It has been demonstrated that the function of RGS16 can be affected at the transcription and post-translation levels, including by induced expression, and modification through phosphorylation and palmitoylation, although this has not been well established and has mostly been reported in cell models, with a lack of studies on specific cells or disease models. RGS16 is expressed in a variety of immune cells and affects the function of these immune cells involved in the development of immune and inflammatory diseases, and this aspect has not been studied in detail. In addition, RGS16 serves a non-negligible role in tumorigenesis, especially in the progression of breast cancer, for which it may be a novel therapeutic target, and its expression levels are also a biomarker for the diagnosis and prognosis of pancreatic cancer, PDA, colorectal cancer and glioma; however, its expression has only been tested in relation to prognosis. In addition, RGS16 is a candidate biomarker for the regulation of central and peripheral biorhythms, and its role has been confirmed in knockout mice; however, the specific mechanism is not clear. Furthermore, it also affects platelet function, and thus, the development of coagulation through non-classical signaling pathways. Finally, although RGS16 is involved in the regulation of classical and other non-classical signaling pathways, its specific interacting molecules and phosphorylation or palmitoylation modification status in these signaling pathways remain to be studied. In conclusion, RGS16 serves an important regulatory role in the development and process of various diseases via GPCRs and other non-classical signaling pathways. Although this has not yet been examined in-depth, it may suggest potential mechanisms and targets for the treatment of these diseases.

## Glossary

GPCRs: G protein-coupled receptors
GDP: Guanosine diphosphate
GEFs: Guanine-nucleotide exchange factors
GTP: Guanosine triphosphate
RGS: Regulators of G protein signaling
GTPase: guanosine triphosphatase
MAPK: mitogen-activated protein kinase
PI3K: phosphoinositide 3-kinase
AKT: protein kinase B
Rho A: Ras homolog family member A
SDF-1: stromal cell-derived factor 1
CXCR4: C-X-C motif chemokine receptor 4 pathways
GAPs: GTPase activating proteins
ERK: extracellular regulated protein kinases
TNFα: tumor necrosis factor α
TLR: toll-like receptor
MHC: major histocompatibility complex
PAR2: protease-activated receptor 2
HER2: human epidermal growth factor receptor-2
EGFR: epidermal growth factor receptor
EMT: epithelial-mesenchymal transition
GFP: green fluorescent protein
PDA: pancreatic ductal adenocarcinoma
HBeAg: hepatitis B e antigen
NF-κB: nuclear factor-κB
cAMP: cyclic adenosine monophosphate
IL: interleukin
LPS: lipopolysaccharide
AHR: airway hyperresponsiveness
GC: germinal center
CXCL12: C-X-C motif chemokine ligand 12
CD4^+^: cluster of differentiation 4-positive
PCV2: porcine circovirus type 2
ORF3: open reading frame 3
SNPs: single nucleotide polymorphisms
SCN: suprachiasmatic nucleus
Gpr176: G protein-coupled receptor 176
ChREBP: carbohydrate response element binding protein
Arg2: arginase 2

## References

[B1] Abramow-Newerly M. Ming H. Chidiac P. (2006). Modulation of subfamily B/R4 RGS protein function by 14-3-3 proteins. Cell. Signal. 18 (12), 2209–2222. 10.1016/j.cellsig.2006.05.011 16839744

[B2] Airoldi I. Raffaghello L. Piovan E. Cocco C. Carlini B. Amadori A. (2006). CXCL12 does not attract CXCR4+ human metastatic neuroblastoma cells: Clinical implications. Clin. Cancer Res. 12 (1), 77–82. 10.1158/1078-0432.CCR-05-1376 16397027

[B3] Almutairi F. Lee J. K. Rada B. (2020). Regulator of G protein signaling 10: Structure, expression and functions in cellular physiology and diseases. Cell. Signal. 75, 109765. 10.1016/j.cellsig.2020.109765 32882407 PMC7579743

[B4] Alqinyah M. Almutairi F. Wendimu M. Y. Hooks S. B. (2018). RGS10 regulates the expression of cyclooxygenase-2 and tumor necrosis factor Alpha through a G protein-independent mechanism. Mol. Pharmacol. 94 (4), 1103–1113. 10.1124/mol.118.111674 30049816 PMC6108573

[B5] Alqinyah M. Hooks S. B. (2018). Regulating the regulators: Epigenetic, transcriptional, and post-translational regulation of RGS proteins. Cell. Signal. 42, 77–87. 10.1016/j.cellsig.2017.10.007 29042285 PMC5732043

[B6] Bai X. Liao Y. Sun F. Xiao X. Fu S. (2021). Diurnal regulation of oxidative phosphorylation restricts hepatocyte proliferation and inflammation. Cell. Rep. 36 (10), 109659. 10.1016/j.celrep.2021.109659 34496251

[B7] Bansal G. Druey K. M. Xie Z. (2007). R4 RGS proteins: Regulation of G-protein signaling and beyond. Pharmacol. Ther. 116 (3), 473–495. 10.1016/j.pharmthera.2007.09.005 18006065 PMC2156173

[B8] Beadling C. Druey K. M. Richter G. Kehrl J. H. Smith K. A. (1999). Regulators of G protein signaling exhibit distinct patterns of gene expression and target G protein specificity in human lymphocytes. J. Immunol. 162 (5), 2677–2682. 10072511

[B9] Berman D. M. Kozasa T. Gilman A. G. (1996). The GTPase-activating protein RGS4 stabilizes the transition state for nucleotide hydrolysis. J. Biol. Chem. 271 (44), 27209–27212. 10.1074/jbc.271.44.27209 8910288

[B10] Berthebaud M. Riviere C. Jarrier P. Foudi A. Zhang Y. Compagno D. (2005). RGS16 is a negative regulator of SDF-1-CXCR4 signaling in megakaryocytes. Blood 106 (9), 2962–2968. 10.1182/blood-2005-02-0526 15998835

[B11] Buckbinder L. Velasco-Miguel S. Chen Y. Xu N. Talbott R. Gelbert L. (1997). The p53 tumor suppressor targets a novel regulator of G protein signaling. Proc. Natl. Acad. Sci. U. S. A. 94 (15), 7868–7872. 10.1073/pnas.94.15.7868 9223279 PMC21521

[B12] Carper M. B. Denvir J. Boskovic G. Primerano D. A. Claudio P. P. (2014). RGS16, a novel p53 and pRb cross-talk candidate inhibits migration and invasion of pancreatic cancer cells. Genes. Cancer 5 (11-12), 420–435. 10.18632/genesandcancer.43 25568667 PMC4279439

[B13] Chan R. K. Otte C. A. (1982). Isolation and genetic analysis of *Saccharomyces cerevisiae* mutants supersensitive to G1 arrest by a factor and alpha factor pheromones. Mol. Cell. Biol. 2 (1), 11–20. 10.1128/mcb.2.1.11-20.1982 7050665 PMC369748

[B14] Chen C. K. Wieland T. Simon M. I. Rgs-r (1996). RGS-r, a retinal specific RGS protein, binds an intermediate conformation of transducin and enhances recycling. Proc. Natl. Acad. Sci. U. S. A. 93 (23), 12885–12889. 10.1073/pnas.93.23.12885 8917514 PMC24015

[B15] Chen C. Lin S. C. (1998). The core domain of RGS16 retains G-protein binding and GAP activity *in vitro*, but is not functional *in vivo* . FEBS Lett. 422 (3), 359–362. 10.1016/s0014-5793(98)00042-8 9498816

[B16] Chen C. Seow K. T. Guo K. Yaw L. P. Lin S. C. (1999). The membrane association domain of RGS16 contains unique amphipathic features that are conserved in RGS4 and RGS5. J. Biol. Chem. 274 (28), 19799–19806. 10.1074/jbc.274.28.19799 10391923

[B17] Chen C. Wang H. Fong C. W. Lin S. C. (2001). Multiple phosphorylation sites in RGS16 differentially modulate its GAP activity. FEBS Lett. 504 (1-2), 16–22. 10.1016/s0014-5793(01)02757-0 11522288

[B18] Chen C. Zheng B. Han J. Lin S. C. (1997). Characterization of a novel mammalian RGS protein that binds to Galpha proteins and inhibits pheromone signaling in yeast. J. Biol. Chem. 272 (13), 8679–8685. 10.1074/jbc.272.13.8679 9079700

[B19] Chen W. Bian H. Xie X. Yang X. Bi B. Li C. (2020). Negative feedback loop of ERK/CREB/miR-212-3p inhibits HBeAg-induced macrophage activation. J. Cell. Mol. Med. 24 (18), 10935–10945. 10.1111/jcmm.15723 32767729 PMC7521245

[B20] Choi C. Y. Rho S. B. Kim H. S. Han J. Bae J. Lee S. J. (2015). The ORF3 protein of porcine circovirus type 2 promotes secretion of IL-6 and IL-8 in porcine epithelial cells by facilitating proteasomal degradation of regulator of G protein signalling 16 through physical interaction. J. Gen. Virol. 96 (5), 1098–1108. 10.1099/vir.0.000046 25575706

[B21] Craft J. E. (2012). Follicular helper T cells in immunity and systemic autoimmunity. Nat. Rev. Rheumatol. 8 (6), 337–347. 10.1038/nrrheum.2012.58 22549246 PMC3604997

[B22] Cunningham M. L. Waldo G. L. Hollinger S. Hepler J. R. Harden T. K. (2001). Protein kinase C phosphorylates RGS2 and modulates its capacity for negative regulation of Galpha 11 signaling. J. Biol. Chem. 276 (8), 5438–5444. 10.1074/jbc.M007699200 11063746

[B23] Davidsson J. Andersson A. Paulsson K. Heidenblad M. Isaksson M. Borg A. (2007). Tiling resolution array comparative genomic hybridization, expression and methylation analyses of dup(1q) in Burkitt lymphomas and pediatric high hyperdiploid acute lymphoblastic leukemias reveal clustered near-centromeric breakpoints and overexpression of genes in 1q22-32.3. Hum. Mol. Genet. 16 (18), 2215–2225. 10.1093/hmg/ddm173 17613536

[B24] De Vries L. Elenko E. Hubler L. Jones T. L. Farquhar M. G. (1996). GAIP is membrane-anchored by palmitoylation and interacts with the activated (GTP-bound) form of G alpha i subunits. Proc. Natl. Acad. Sci. U. S. A. 93 (26), 15203–15208. 10.1073/pnas.93.26.15203 8986788 PMC26381

[B25] De Vries L. Gist Farquhar M. (1999). RGS proteins: More than just GAPs for heterotrimeric G proteins. Trends Cell. Biol. 9 (4), 138–144. 10.1016/s0962-8924(99)01515-9 10203790

[B26] Derrien A. Druey K. M. (2001). RGS16 function is regulated by epidermal growth factor receptor-mediated tyrosine phosphorylation. J. Biol. Chem. 276 (51), 48532–48538. 10.1074/jbc.M108862200 11602604

[B27] Derrien A. Zheng B. Osterhout J. L. Ma Y. C. Milligan G. Farquhar M. G. (2003). Src-mediated RGS16 tyrosine phosphorylation promotes RGS16 stability. J. Biol. Chem. 278 (18), 16107–16116. 10.1074/jbc.M210371200 12588871

[B28] Ding Y. Li J. Wu Q. Yang P. Luo B. Xie S. (2013). IL-17RA is essential for optimal localization of follicular Th cells in the germinal center light zone to promote autoantibody-producing B cells. J. Immunol. 191 (4), 1614–1624. 10.4049/jimmunol.1300479 23858031 PMC3819396

[B29] Dohlman H. G. Song J. Ma D. Courchesne W. E. Thorner J. (1996). Sst2, a negative regulator of pheromone signaling in the yeast *Saccharomyces cerevisiae*: Expression, localization, and genetic interaction and physical association with Gpa1 (the G-protein alpha subunit). Mol. Cell. Biol. 16 (9), 5194–5209. 10.1128/MCB.16.9.5194 8756677 PMC231520

[B30] Doi M. Murai I. Kunisue S. Setsu G. Uchio N. Tanaka R. (2016). Gpr176 is a Gz-linked orphan G-protein-coupled receptor that sets the pace of circadian behaviour. Nat. Commun. 7, 10583. 10.1038/ncomms10583 26882873 PMC4757782

[B31] Druey K. M. Ugur O. Caron J. M. Chen C. K. Backlund P. S. Jones T. L. (1999). Amino-terminal cysteine residues of RGS16 are required for palmitoylation and modulation of Gi- and Gq-mediated signaling. J. Biol. Chem. 274 (26), 18836–18842. 10.1074/jbc.274.26.18836 10373502

[B32] DʼSouza M. S. Guisinger T. C. Norman H. Seeley S. L. Chrissobolis S. (2019). Regulator of G-protein signaling 5 protein protects against anxiety- and depression-like behavior. Behav. Pharmacol. 30 (8), 712–721. 10.1097/FBP.0000000000000506 31625976

[B33] Du Y. S. Huang B. R. (2005). The structure, classification and function of RGS proteins. Sheng Li Ke Xue Jin Zhan 36 (3), 215–219. 16270819

[B34] Estes J. D. Thacker T. C. Hampton D. L. Kell S. A. Keele B. F. Palenske E. A. (2004). Follicular dendritic cell regulation of CXCR4-mediated germinal center CD4 T cell migration. J. Immunol. 173 (10), 6169–6178. 10.4049/jimmunol.173.10.6169 15528354

[B35] Faurobert E. Hurley J. B. (1997). The core domain of a new retina specific RGS protein stimulates the GTPase activity of transducin *in vitro* . Proc. Natl. Acad. Sci. U. S. A. 94 (7), 2945–2950. 10.1073/pnas.94.7.2945 9096326 PMC20302

[B36] Faurobert E. Scotti A. Hurley J. B. Chabre M. (1999). RET-RGS, a retina-specific regulator of G-protein signaling, is located in synaptic regions of the rat retina. Neurosci. Lett. 269 (1), 41–44. 10.1016/s0304-3940(99)00423-1 10821640

[B37] Gao Y. Hu H. Ramachandran S. Erickson J. W. Cerione R. A. Skiniotis G. (2019). Structures of the rhodopsin-transducin complex: Insights into G-protein activation. Mol. Cell. 75 (4), 781–790.e3. e3. 10.1016/j.molcel.2019.06.007 31300275 PMC6707884

[B38] Gilman A. G. (1987). G proteins: Transducers of receptor-generated signals. Annu. Rev. Biochem. 56, 615–649. 10.1146/annurev.bi.56.070187.003151 3113327

[B39] Grafstein-Dunn E. Young K. H. Cockett M. I. Khawaja X. Z. (2001). Regional distribution of regulators of G-protein signaling (RGS) 1, 2, 13, 14, 16, and GAIP messenger ribonucleic acids by *in situ* hybridization in rat brain. Brain Res. Mol. Brain Res. 88 (1-2), 113–123. 10.1016/s0169-328x(01)00038-9 11295237

[B40] Gu S. Cifelli C. Wang S. Heximer S. P. (2009). RGS proteins: Identifying new GAPs in the understanding of blood pressure regulation and cardiovascular function. Clin. Sci. 116 (5), 391–399. 10.1042/CS20080272 19175357

[B41] Hayasaka N. Aoki K. Kinoshita S. Yamaguchi S. Wakefield J. K. Tsuji-Kawahara S. (2017). Attenuated food anticipatory activity and abnormal circadian locomotor rhythms in Rgs16 knockdown mice. J. PLoS ONE 6 (3), e17655. 10.1371/journal.pone.0017655 PMC3052372 21408016

[B42] Hayasaka N. Aoki K. Kinoshita S. Yamaguchi S. Wakefield J. K. Tsuji-Kawahara S. (2011). Attenuated food anticipatory activity and abnormal circadian locomotor rhythms in Rgs16 knockdown mice. PLoS One 6 (3), e17655. 10.1371/journal.pone.0017655 21408016 PMC3052372

[B43] Hendriks-Balk M. C. Peters S. L. M. Michel M. C. Alewijnse A. E. (2008). Regulation of G protein-coupled receptor signalling: Focus on the cardiovascular system and regulator of G protein signalling proteins. Eur. J. Pharmacol. 585 (2-3), 278–291. 10.1016/j.ejphar.2008.02.088 18410914

[B44] Hernandez K. R. Karim Z. A. Qasim H. Druey K. M. Alshbool F. Z. Khasawneh F. T. (2019). Regulator of G-protein signaling 16 is a negative modulator of platelet function and thrombosis. J. Am. Heart Assoc. 8 (5), e011273. 10.1161/JAHA.118.011273 30791801 PMC6474914

[B45] Heximer S. P. Watson N. Linder M. E. Blumer K. J. Hepler J. R. (1997). RGS2/G0S8 is a selective inhibitor of Gqalpha function. Proc. Natl. Acad. Sci. U. S. A. 94 (26), 14389–14393. 10.1073/pnas.94.26.14389 9405622 PMC24991

[B46] Hooks S. B. Waldo G. L. Corbitt J. Bodor E. T. Krumins A. M. Harden T. K. (2003). RGS6, RGS7, RGS9, and RGS11 stimulate GTPase activity of Gi family G-proteins with differential selectivity and maximal activity. J. Biol. Chem. 278 (12), 10087–10093. 10.1074/jbc.M211382200 12531899

[B47] Hoshi Y. Endo K. Shirakihara T. Fukagawa A. Miyazawa K. Saitoh M. (2016). The potential role of regulator of G-protein signaling 16 in cell motility mediated by δEF1 family proteins. FEBS Lett. 590 (2), 270–278. 10.1002/1873-3468.12042 26823172 PMC4819697

[B48] Hsu H. C. Yang P. Wang J. Wu Q. Myers R. Chen J. (2008). Interleukin 17-producing T helper cells and interleukin 17 orchestrate autoreactive germinal center development in autoimmune BXD2 mice. Nat. Immunol. 9 (2), 166–175. 10.1038/ni1552 18157131

[B49] Hu X. Tang J. Zeng G. Bao P. Wu J. (2019). RGS1 silencing inhibits the inflammatory response and angiogenesis in rheumatoid arthritis rats through the inactivation of Toll-like receptor signaling pathway. J. Cell. Physiol. 234 (11), 20432–20442. 10.1002/jcp.28645 31012109

[B50] Hu Y. Zheng M. Wang S. Gao L. Gou R. Liu O. (2021). Identification of a five-gene signature of the RGS gene family with prognostic value in ovarian cancer. Genomics 113 (4), 2134–2144. 10.1016/j.ygeno.2021.04.012 33845140

[B51] Huang J. Pashkov V. Kurrasch D. M. Yu K. Gold S. J. Wilkie T. M. (2006). Feeding and fasting controls liver expression of a regulator of G protein signaling (Rgs16) in periportal hepatocytes. Comp. Hepatol. 5, 8. 10.1186/1476-5926-5-8 17123436 PMC1687201

[B52] Huang R. Li G. Zhao Z. Zeng F. Zhang K. Liu Y. (2020). RGS16 promotes glioma progression and serves as a prognostic factor. CNS Neurosci. Ther. 26 (8), 791–803. 10.1111/cns.13382 32319728 PMC7366748

[B53] Johnson E. N. Seasholtz T. M. Waheed A. A. Kreutz B. Suzuki N. Kozasa T. (2003). RGS16 inhibits signalling through the G alpha 13-Rho axis. Nat. Cell. Biol. 5 (12), 1095–1103. 10.1038/ncb1065 14634662

[B54] Karim Z. A. Alshbool F. Z. Vemana H. P. Conlon C. Druey K. M. Khasawneh F. T. (2016). CXCL12 regulates platelet activation via the regulator of G-protein signaling 16. Biochim. Biophys. Acta 1863 (2), 314–321. 10.1016/j.bbamcr.2015.11.028 26628381 PMC10983798

[B55] Kim J. H. Lee J. Y. Lee K. T. Lee J. K. Lee K. H. Jang K. T. (2010). RGS16 and FosB underexpressed in pancreatic cancer with lymph node metastasis promote tumor progression. Tumour Biol. 31 (5), 541–548. 10.1007/s13277-010-0067-z 20571966

[B56] Kim K. Lee J. Ghil S. (2018). The regulators of G protein signaling RGS16 and RGS18 inhibit protease-activated receptor 2/Gi/o signaling through distinct interactions with Gα in live cells. FEBS Lett. 592 (18), 3126–3138. 10.1002/1873-3468.13220 30117167

[B57] Kim S. D. Sung H. J. Park S. K. Kim T. W. Park S. C. (2006). The expression patterns of RGS transcripts in platelets. Platelets 17 (7), 493–497. 10.1080/09537100600758123 17074726

[B58] Kimple A. J. Bosch D. E. Giguere P. M. Siderovski D. P. (2011). Regulators of G-protein signaling and their Gα substrates: Promises and challenges in their use as drug discovery targets. Pharmacol. Rev. 63 (3), 728–749. 10.1124/pr.110.003038 21737532 PMC3141876

[B59] Koelle M. R. (1997). A new family of G-protein regulators - the RGS proteins. Curr. Opin. Cell. Biol. 9 (2), 143–147. 10.1016/s0955-0674(97)80055-5 9069252

[B60] Koelle M. R. Horvitz H. R. (1996). EGL-10 regulates G protein signaling in the *C. elegans* nervous system and shares a conserved domain with many mammalian proteins. Cell. 84 (1), 115–125. 10.1016/s0092-8674(00)80998-8 8548815

[B61] Kozasa T. Jiang X. Hart M. J. Sternweis P. M. Singer W. D. Gilman A. G. (1998). p115 RhoGEF, a GTPase activating protein for Galpha12 and Galpha13. Science 280 (5372), 2109–2111. 10.1126/science.280.5372.2109 9641915

[B62] Krieger N. S. Bushinsky D. A. (2021). Metabolic acidosis regulates RGS16 and G protein signaling in osteoblasts. Am. J. Physiol. Ren. Physiol. 321 (4), F424–f430. 10.1152/ajprenal.00166.2021 PMC8560403 34396788

[B63] Kveberg L. Ryan J. C. Rolstad B. Inngjerdingen M. (2005). Expression of regulator of G protein signalling proteins in natural killer cells, and their modulation by Ly49A and Ly49D. Immunology 115 (3), 358–365. 10.1111/j.1365-2567.2005.02174.x 15946253 PMC1782169

[B64] Lambrecht B. N. Hammad H. (2015). The immunology of asthma. Nat. Immunol. 16 (1), 45–56. 10.1038/ni.3049 25521684

[B65] Layeghi-Ghalehsoukhteh S. Pal Choudhuri S. Ocal O. Zolghadri Y. Pashkov V. Niederstrasser H. (2020). Concerted cell and *in vivo* screen for pancreatic ductal adenocarcinoma (PDA) chemotherapeutics. Sci. Rep. 10 (1), 20662. 10.1038/s41598-020-77373-8 33244070 PMC7693321

[B66] Lee S. H. Lim K. S. Hong K. C. Kim J. M. (2021). Genetic association of polymorphisms in porcine RGS16 with porcine circovirus viral load in naturally infected Yorkshire pigs. J. Anim. Sci. Technol. 63 (6), 1223–1231. 10.5187/jast.2021.e105 34957439 PMC8672253

[B67] Liang G. Bansal G. Xie Z. Druey K. M. (2009). RGS16 inhibits breast cancer cell growth by mitigating phosphatidylinositol 3-kinase signaling. J. Biol. Chem. 284 (32), 21719–21727. 10.1074/jbc.M109.028407 19509421 PMC2755894

[B68] Linterman M. A. Rigby R. J. Wong R. K. Yu D. Brink R. Cannons J. L. (2009). Follicular helper T cells are required for systemic autoimmunity. J. Exp. Med. 206 (3), 561–576. 10.1084/jem.20081886 19221396 PMC2699132

[B69] Lippert E. Yowe D. L. Gonzalo J. A. Justice J. P. Webster J. M. Fedyk E. R. (2003). Role of regulator of G protein signaling 16 in inflammation-induced T lymphocyte migration and activation. J. Immunol. 171 (3), 1542–1555. 10.4049/jimmunol.171.3.1542 12874248

[B70] Liu T. Bohlken A. Kuljaca S. Lee M. Nguyen T. Smith S. (2005). The retinoid anticancer signal: Mechanisms of target gene regulation. Br. J. Cancer 93 (3), 310–318. 10.1038/sj.bjc.6602700 16012519 PMC2361573

[B71] Masuho I. Balaji S. Muntean B. S. Skamangas N. K. Chavali S. Tesmer J. J. G. (2020). A global map of G protein signaling regulation by RGS proteins. Cell. 183 (2), 503–521.e19. e19. 10.1016/j.cell.2020.08.052 33007266 PMC7572916

[B72] Mesin L. Ersching J. Victora G. D. (2016). Germinal center B cell dynamics. Immunity 45 (3), 471–482. 10.1016/j.immuni.2016.09.001 27653600 PMC5123673

[B73] Mieda M. Ono D. Hasegawa E. Okamoto H. Honma K. I. Honma S. (2015). Cellular clocks in AVP neurons of the SCN are critical for interneuronal coupling regulating circadian behavior rhythm. Neuron 85 (5), 1103–1116. 10.1016/j.neuron.2015.02.005 25741730

[B74] Miyoshi N. Ishii H. Sekimoto M. Doki Y. Mori M. (2009). RGS16 is a marker for prognosis in colorectal cancer. Ann. Surg. Oncol. 16 (12), 3507–3514. 10.1245/s10434-009-0690-3 19760045

[B75] Muma N. A. (2012). RGS proteins: Impact on the treatment of depression and anxiety. Int. J. Neuropsychopharmacol. 15 (9), 1199–1200. 10.1017/S1461145711002008 22277123

[B76] Neves S. R. Ram P. T. Iyengar R. (2002). G protein pathways. Science 296 (5573), 1636–1639. 10.1126/science.1071550 12040175

[B77] O'Brien J. B. Wilkinson J. C. Roman D. L. (2019). Regulator of G-protein signaling (RGS) proteins as drug targets: Progress and future potentials. J. Biol. Chem. 294 (49), 18571–18585. 10.1074/jbc.REV119.007060 31636120 PMC6901330

[B78] Ocal O. Pashkov V. Kollipara R. K. Zolghadri Y. Cruz V. H. Hale M. A. (2015). A rapid *in vivo* screen for pancreatic ductal adenocarcinoma therapeutics. Dis. Model. Mech. 8 (10), 1201–1211. 10.1242/dmm.020933 26438693 PMC4610235

[B79] Perrier P. Martinez F. O. Locati M. Bianchi G. Nebuloni M. Vago G. (2004). Distinct transcriptional programs activated by interleukin-10 with or without lipopolysaccharide in dendritic cells: Induction of the B cell-activating chemokine, CXC chemokine ligand 13. J. Immunol. 172 (11), 7031–7042. 10.4049/jimmunol.172.11.7031 15153525

[B80] Rendu F. Brohard-Bohn B. (2001). The platelet release reaction: Granules' constituents, secretion and functions. Platelets 12 (5), 261–273. 10.1080/09537100120068170 11487378

[B81] Richman R. W. Strock J. Hains M. D. Cabanilla N. J. Lau K. K. Siderovski D. P. (2005). RGS12 interacts with the SNARE-binding region of the Cav2.2 calcium channel. J. Biol. Chem. 280 (2), 1521–1528. 10.1074/jbc.M406607200 15536086

[B82] Roh J. Shin S. J. Lee A. N. Yoon D. H. Suh C. Park C. J. (2017). RGS1 expression is associated with poor prognosis in multiple myeloma. J. Clin. Pathol. 70 (3), 202–207. 10.1136/jclinpath-2016-203713 27445341

[B83] Ross D. W. Ayscue L. H. Watson J. Bentley S. A. (1988). Stability of hematologic parameters in healthy subjects. Intraindividual versus interindividual variation. Am. J. Clin. Pathol. 90 (3), 262–267. 10.1093/ajcp/90.3.262 3414599

[B84] Ross E. M. Wilkie T. M. (2000). GTPase-activating proteins for heterotrimeric G proteins: regulators of G protein signaling (RGS) and RGS-like proteins. Annu. Rev. Biochem. 69, 795–827. 10.1146/annurev.biochem.69.1.795 10966476

[B85] Sae-Lee C. Moolsuwan K. Chan L. Poungvarin N. (2016). ChREBP regulates itself and metabolic genes implicated in lipid accumulation in β-cell line. PLoS One 11 (1), e0147411. 10.1371/journal.pone.0147411 26808438 PMC4725739

[B86] Sambi B. S. Hains M. D. Waters C. M. Connell M. C. Willard F. S. Kimple A. J. (2006). The effect of RGS12 on PDGFbeta receptor signalling to p42/p44 mitogen activated protein kinase in mammalian cells. Cell. Signal. 18 (7), 971–981. 10.1016/j.cellsig.2005.08.003 16214305

[B87] Schiff M. L. Siderovski D. P. Jordan J. D. Brothers G. Snow B. De Vries L. (2000). Tyrosine-kinase-dependent recruitment of RGS12 to the N-type calcium channel. Nature 408 (6813), 723–727. 10.1038/35047093 11130074

[B88] Shankar S. P. Wilson M. S. DiVietro J. A. Mentink-Kane M. M. Xie Z. Wynn T. A. (2012). RGS16 attenuates pulmonary Th2/Th17 inflammatory responses. J. Immunol. 188 (12), 6347–6356. 10.4049/jimmunol.1103781 22593615 PMC3522182

[B89] Shi G. X. Harrison K. Han S. B. Moratz C. Kehrl J. H. (2004). Toll-like receptor signaling alters the expression of regulator of G protein signaling proteins in dendritic cells: implications for G protein-coupled receptor signaling. J. Immunol. 172 (9), 5175–5184. 10.4049/jimmunol.172.9.5175 15100254

[B90] Shu F. J. Ramineni S. Hepler J. R. (2010). RGS14 is a multifunctional scaffold that integrates G protein and Ras/Raf MAPkinase signalling pathways. Cell. Signal. 22 (3), 366–376. 10.1016/j.cellsig.2009.10.005 19878719 PMC2795083

[B91] Siehler S. (2009). Regulation of RhoGEF proteins by G12/13-coupled receptors. Br. J. Pharmacol. 158 (1), 41–49. 10.1111/j.1476-5381.2009.00121.x 19226283 PMC2795247

[B92] Sierra D. A. Gilbert D. J. Householder D. Grishin N. V. Yu K. Ukidwe P. (2002). Evolution of the regulators of G-protein signaling multigene family in mouse and human. Genomics 79 (2), 177–185. 10.1006/geno.2002.6693 11829488

[B93] Sjögren B. Blazer L. L. Neubig R. R. (2010). Regulators of G protein signaling proteins as targets for drug discovery. Prog. Mol. Biol. Transl. Sci. 91, 81–119. 10.1016/S1877-1173(10)91004-1 20691960

[B94] Slep K. C. Kercher M. A. Wieland T. Chen C. K. Simon M. I. Sigler P. B. (2008). Molecular architecture of Galphao and the structural basis for RGS16-mediated deactivation. Proc. Natl. Acad. Sci. U. S. A. 105 (17), 6243–6248. 10.1073/pnas.0801569105 18434540 PMC2359805

[B95] Snow B. E. Antonio L. Suggs S. Siderovski D. P. (1998). Cloning of a retinally abundant regulator of G-protein signaling (RGS-r/RGS16): Genomic structure and chromosomal localization of the human gene. Gene 206 (2), 247–253. 10.1016/s0378-1119(97)00593-3 9469939

[B96] Snow B. E. Hall R. A. Krumins A. M. Brothers G. M. Bouchard D. Brothers C. A. (1998). GTPase activating specificity of RGS12 and binding specificity of an alternatively spliced PDZ (PSD-95/Dlg/ZO-1) domain. J. Biol. Chem. 273 (28), 17749–17755. 10.1074/jbc.273.28.17749 9651375

[B97] Soundararajan M. Willard F. S. Kimple A. J. Turnbull A. P. Ball L. J. Schoch G. A. (2008). Structural diversity in the RGS domain and its interaction with heterotrimeric G protein alpha-subunits. Proc. Natl. Acad. Sci. U. S. A. 105 (17), 6457–6462. 10.1073/pnas.0801508105 18434541 PMC2359823

[B98] Sun X. Charbonneau C. Wei L. Chen Q. Terek R. M. (2015). miR-181a targets RGS16 to promote chondrosarcoma growth, angiogenesis, and metastasis. Mol. Cancer Res. 13 (9), 1347–1357. 10.1158/1541-7786.MCR-14-0697 26013170 PMC4573256

[B99] Suurväli J. Pahtma M. Saar R. Paalme V. Nutt A. Tiivel T. (2015). RGS16 restricts the pro-inflammatory response of monocytes. Scand. J. Immunol. 81 (1), 23–30. 10.1111/sji.12250 25366993

[B100] Suurväli J. Robert J. Boudinot P. Ruutel Boudinot S. (2013). R4 regulators of G protein signaling (RGS) identify an ancient MHC-linked synteny group. Immunogenetics 65 (2), 145–156. 10.1007/s00251-012-0661-x 23129146 PMC3545050

[B101] Syrovatkina V. Alegre K. O. Dey R. Huang X. Y. (2016). Regulation, signaling, and physiological functions of G-proteins. J. Mol. Biol. 428 (19), 3850–3868. 10.1016/j.jmb.2016.08.002 27515397 PMC5023507

[B102] Tany R. Goto Y. Kondo Y. Aoki K. (2022). Quantitative live-cell imaging of GPCR downstream signaling dynamics. Biochem. J. 479 (8), 883–900. 10.1042/BCJ20220021 35383830

[B103] Timmusk S. Merlot E. Lovgren T. Jarvekulg L. Berg M. Fossum C. (2009). Regulator of G protein signalling 16 is a target for a porcine circovirus type 2 protein. J. Gen. Virol. 90 (10), 2425–2436. 10.1099/vir.0.008896-0 19570954

[B104] Ueda H. R. Chen W. Adachi A. Wakamatsu H. Hayashi S. Takasugi T. (2002). A transcription factor response element for gene expression during circadian night. Nature 418 (6897), 534–539. 10.1038/nature00906 12152080

[B105] Villasenor A. Wang Z. V. Rivera L. B. Ocal O. Asterholm I. W. Scherer P. E. (2010). Rgs16 and Rgs8 in embryonic endocrine pancreas and mouse models of diabetes. Dis. Model. Mech. 3 (9-10), 567–580. 10.1242/dmm.003210 20616094 PMC2931535

[B106] Vivot K. Moulle V. S. Zarrouki B. Tremblay C. Mancini A. D. Maachi H. (2016). The regulator of G-protein signaling RGS16 promotes insulin secretion and β-cell proliferation in rodent and human islets. Mol. Metab. 5 (10), 988–996. 10.1016/j.molmet.2016.08.010 27689011 PMC5034687

[B107] Walker L. S. Wiggett H. E. Gaspal F. M. C. Raykundalia C. R. Goodall M. D. Toellner K. M. (2003). Established T cell-driven germinal center B cell proliferation is independent of CD28 signaling but is tightly regulated through CTLA-4. J. Immunol. 170 (1), 91–98. 10.4049/jimmunol.170.1.91 12496387

[B108] Walsh T. G. Harper M. T. Poole A. W. (2015). SDF-1α is a novel autocrine activator of platelets operating through its receptor CXCR4. Cell. Signal. 27 (1), 37–46. 10.1016/j.cellsig.2014.09.021 25283599 PMC4265729

[B109] Wang D. Yu H. Zhou X. Liu X. Zhang R. Lu Z. (2019). Author Correction: Probing the crystallographic orientation of two-dimensional atomic crystals with supramolecular self-assembly. Nat. Commun. 41 (1), 165–177. 10.1038/s41467-018-08077-x PMC6325241 30622269

[B110] Wang J. Ducret A. Tu Y. Kozasa T. Aebersold R. Ross E. M. (1998). RGSZ1, a Gz-selective RGS protein in brain. Structure, membrane association, regulation by Galphaz phosphorylation, and relationship to a Gz gtpase-activating protein subfamily. J. Biol. Chem. 273 (40), 26014–26025. 10.1074/jbc.273.40.26014 9748280

[B111] Wang J. Hua T. Liu Z. J. (2020). Structural features of activated GPCR signaling complexes. Curr. Opin. Struct. Biol. 63, 82–89. 10.1016/j.sbi.2020.04.008 32485565

[B112] Wang Y. Ho G. Zhang J. J. Nieuwenhuijsen B. Edris W. Chanda P. K. (2002). Regulator of G protein signaling Z1 (RGSZ1) interacts with Galpha i subunits and regulates Galpha i-mediated cell signaling. J. Biol. Chem. 277 (50), 48325–48332. 10.1074/jbc.M206116200 12379657

[B113] Weisshaar N. Wu J. Ming Y. Madi A. Hotz-Wagenblatt A. Ma S. (2022). Rgs16 promotes antitumor CD8(+) T cell exhaustion. Sci. Immunol. 7 (71), eabh1873. 10.1126/sciimmunol.abh1873 35622904

[B114] Wiechec E. Overgaard J. Hansen L. L. (2008). A fragile site within the HPC1 region at 1q25.3 affecting RGS16, RGSL1, and RGSL2 in human breast carcinomas. Genes. Chromosom. Cancer 47 (9), 766–780. 10.1002/gcc.20578 18521847

[B115] Wiechec E. Wiuf C. Overgaard J. Hansen L. L. (2011). High-resolution melting analysis for mutation screening of RGSL1, RGS16, and RGS8 in breast cancer. Cancer Epidemiol. Biomarkers Prev. 20 (2), 397–407. 10.1158/1055-9965.EPI-10-0514 21135262

[B116] Willard F. S. Willard M. D. Kimple A. J. Soundararajan M. Oestreich E. A. Li X. (2009). Regulator of G-protein signaling 14 (RGS14) is a selective H-Ras effector. PLoS One 4 (3), e4884. 10.1371/journal.pone.0004884 19319189 PMC2655719

[B117] Xie S. Li J. Wang J. H. Wu Q. Yang P. Hsu H. C. (2010). IL-17 activates the canonical NF-kappaB signaling pathway in autoimmune B cells of BXD2 mice to upregulate the expression of regulators of G-protein signaling 16. J. Immunol. 184 (5), 2289–2296. 10.4049/jimmunol.0903133 20139273 PMC2849003

[B118] Xie X. Lv H. Liu C. Su X. Yu Z. Song S. (2021). HBeAg mediates inflammatory functions of macrophages by TLR2 contributing to hepatic fibrosis. BMC Med. 19 (1), 247. 10.1186/s12916-021-02085-3 34649530 PMC8518250

[B119] Xie Z. Chan E. C. Druey K. M. (2016). R4 regulator of G protein signaling (RGS) proteins in inflammation and immunity. Aaps J. 18 (2), 294–304. 10.1208/s12248-015-9847-0 26597290 PMC4779105

[B120] Yowe D. Weich N. Prabhudas M. Poisson L. Errada P. Kapeller R. (2001). RGS18 is a myeloerythroid lineage-specific regulator of G-protein-signalling molecule highly expressed in megakaryocytes. Biochem. J. 359 (1), 109–118. 10.1042/0264-6021:3590109 11563974 PMC1222126

[B121] Zhang Y. Higgins C. B. Fortune H. M. Chen P. Stothard A. I. Mayer A. L. (2019). Hepatic arginase 2 (Arg2) is sufficient to convey the therapeutic metabolic effects of fasting. Nat. Commun. 10 (1), 1587. 10.1038/s41467-019-09642-8 30962478 PMC6453920

[B122] Zhang Y. Neo S. Y. Han J. Yaw L. P. Lin S. C. (1999). RGS16 attenuates galphaq-dependent p38 mitogen-activated protein kinase activation by platelet-activating factor. J. Biol. Chem. 274 (5), 2851–2857. 10.1074/jbc.274.5.2851 9915820

[B123] Zheng B. Chen D. Farquhar M. G. (2000). MIR16, a putative membrane glycerophosphodiester phosphodiesterase, interacts with RGS16. Proc. Natl. Acad. Sci. U. S. A. 97 (8), 3999–4004. 10.1073/pnas.97.8.3999 10760272 PMC18131

[B124] Zolghadri Y. Pal Choudhuri S. Ocal O. Layeghi-Ghalehsoukhteh S. Berhe F. Hale M. A. (2018). Malnutrition in pancreatic ductal adenocarcinoma (PDA): Dietary pancreatic enzymes improve short-term health but stimulate tumor growth. Am. J. Pathol. 188 (3), 616–626. 10.1016/j.ajpath.2017.11.014 29248457 PMC5842033

